# Where in the brain is creativity? The fallacy of a creativity faculty in the brain

**DOI:** 10.3389/fpsyg.2024.1373299

**Published:** 2024-04-30

**Authors:** Arne Dietrich

**Affiliations:** Department of Psychology, American University of Beirut, Beirut, Lebanon

**Keywords:** alternative uses test, consciousness, divergent thinking, multiple realizability, neuroscience, default mode network, reification, neuroimaging

## Abstract

The neuroscience of creativity is built on a tacit and near universal assumption that is false. Paradoxically, this is not contentious; once made explicit, the assumption is readily conceded as false. Psychology regards creativity as made up of many complex, multifaceted, and varied cognitive and emotional processes deployed across many different domains. But we instead think of, and treat, creativity as if it were a single, separate, cohesive, and discrete thing—as in, Einstein had *it*. In a straightforward extension of this fallacy, cognitive neuroscientists have looked for uniquely creative cognition that (1) is distinct from all other kinds of cognition and (2) has a proprietary neural substrate. In other words, a standalone and monolithic creativity faculty in the brain that manages *only* creativity and *all* creativity. First, this paper brings into sharp focus the nature and ubiquity of this fallacy. It then outlines the alternative theoretical position that is (1) based on fundamental neural principles and (2) predicated on taking seriously the concept of creativity as complex and diverse. Like morality or secretiveness, it holds that creativity does not exist as its own, specialized entity in the brain. Instead, its neurocognitive mechanisms are distributed, embedded, and varied; that is, creativity is everywhere and multiply realizable.

## 1 Introduction

Consider a few soundbites that could have been overheard in a local bar, business meeting, or discussion among friends: “Creativity is associated with intrinsic curiosity and playfulness; creative people are more sensitive and observant; they tolerate ambiguity better and maintain a child-like naiveté; creativity is enhanced by mindfulness, exercise, and keeping an open mind; creative thinking needs a positive attitude and the ability to perceive complex patterns; there is a thin line between madness and creative genius; creative personalities are not afraid of taking risks; they are often chaotic and rebellious but at the same time self-critical and reflective; mindwandering facilitates creative insights in the unconscious mind.”

To forestall any impression that the present article targets claims circulating in the general public, consider a sampler from recent neuroscience articles: “… the ability to generate creative ideas is characterized by increased functional connectivity between the inferior prefrontal cortex and the default network …” (Beaty et al., 2014). “*… the highly creative* group utilized bilateral prefrontal regions when doing the Brick task, while the *low creative group* used functions predominantly on the left side” (Carlsson et al., 2000). “But, once the brain is sufficiently focused, the cortex needs to relax in order to seek out the more remote association in the right hemisphere, which will provide the insight” (Lehrer, 2008). “EEG Alpha power increases during creative ideation …” (Fink and Benedek, 2014). “… insight culminates with a sharp increase in neural activity in the right anterior temporal lobe at the moment of insight” (Kounios and Beeman, 2014). “… when participants were being creative, as opposed to uncreative, there was an increase in activity in the prefrontal areas, including bilateral medial frontal gyri and left anterior cingulate cortex (ACC)” (Howard-Jones et al., 2005). “… general, creative idea generation (i.e., divergent thinking) was associated with extended activations in the left prefrontal cortex and the right medial temporal lobe, and with deactivation of the right temporoparietal junction.” … “We conclude that the process of idea generation can be generally understood as a state of focused internally-directed attention involving controlled semantic retrieval” (Benedek et al., 2014). “… individual creativity, as measured by the divergent thinking test, is mainly related to the regional gray matter of brain regions known to be associated with the dopaminergic system, congruent with the idea that dopaminergic physiological mechanisms are associated with individual creativity” (Takeuchi et al., 2010). “A region of left frontopolar cortex, previously associated with creative integration of semantic information, exhibited increased activity and functional connectivity to anterior cingulate gyrus and right frontopolar cortex during cued augmentation of state creativity” (Green et al., 2015).

While one might be tempted to quibble with one or the other specific claim, this article submits that they are all false, along with a seemingly infinite number of other such proclamations about the nature of creativity.

## 2 The creativity faculty

So, where is the error here? There are, in fact, two separate errors, made in succession. The first is the creativity faculty fallacy, the mistaken thinking that creativity is its own and unified thing. The second is the false category formation, the habit of prematurely linking this creativity faculty, in its entirety, to one side of a specific ability, characteristic, trait, behavior, mental process, or neural system, despite evidence that creative acts can just as well come into existence otherwise (Dietrich, 2015, 2019a).

Note that these opening statements are all universal claims, made about creativity as a whole. In consequence, they inherently contain two assumptions about the nature of creativity: that it is (1) an independent and (2) a homogeneous entity. The false category formation is a claim about how this entity then relates to other phenomena, a matter briefly highlighted in a later section. This current section focuses on bringing to the fore the tacit assumption of a standalone and monolithic creativity faculty, because any lack of clarity here, at the level of the psychological construct, is prone to lead to mistakes once it is applied to neuroscience.

In a view approaching unanimity, psychology regards creativity as made up of many complex, multifaceted, and varied cognitive and emotional processes deployed across many different domains (e.g., Torrance, 1974; Weisberg, 1993; Runco, 1999; Ward et al., 1999; Dietrich, 2004; Abraham, 2013, 2018; Baer, 2016). What scientists, designers, artists, engineers, entrepreneurs, or ballet dancers do to be creative in their respective spheres are so distinct that these varied activities cannot be subsumed under the category of “creativity.” Creative behavior manifests itself in the human population in such a variety of ways that its underlying cognitive and neural processes must necessarily be very diverse.

Despite this appreciation of the complexity and diversity of creativity at the theoretical level, this is not how we think of, and treat, creativity in practice, either in the public arena or in the professional field. Instead, we conceive of, and empirical investigate, creativity as if it were a single, separate, cohesive, and discrete thing. In personality and social psychology, for instance, it is its own character trait that exists apart from all other traits (e.g., Amabile, 1983; Eysenck, 1993; Gardner, 1993; Csikszentmihalyi, 1996). Steve Jobs, for instance, was considered creative. The claim is not that he had a part, aspect, feature, or type of creativity, nor is his innovative output understood in terms of a mix of other qualities, processes, or abilities.

In cognitive psychology, the explicit rationale of empirical work is to investigate creativity head-on, as a sovereign cognitive unit in its own right and with its own boundaries (e.g., Guilford, 1967; Smith et al., 1995; Boden, 1998; Runco, 2004). This rationale is reflected in the experimental methods.

Following our folk psychology understanding of creativity, early theories in the 50s and 60s presumed that creativity is an autonomous, domain-general capacity or talent. This was operationalized with the notion of divergent thinking, defined as the ability to generate multiple solutions to an open-ended problem (Guilford, 1950, 1967), which led to the subsequent development of several standardized psychometric instruments, such as the Remote Associates Test (RAT; Mednick, 1962) or the Torrance Test of Creative Thinking (TTCT; Torrance, 1974). While the general-capacity theories have been replaced in favor of the view that creativity is complex, multifaceted, and varied, divergent thinking tests have thrived and come to dominate the experimental approach to creativity research, most likely because they are easy to use and readily available (Baer, 2022).

Bracketing for now that these ‘creativity tests’ have been shown for over half a century to lack ecological validity, certainly in the severely shortened version used for neuroimaging (e.g., Thorndike, 1963; Crockenberg, 1972; Wallach, 1976; Sternberg, 1985; Plucker, 1999; Ward et al., 1999; Kerr and Gagliardi, 2003; Silvia et al., 2008; Arden et al., 2010; Dietrich and Kanso, 2010; Sawyer, 2012; Abraham, 2013; Weisberg, 2013; Baer, 2016 for a detailed overview, see Baer, 2022), the divergent thinking paradigm is unambiguously committed to a creativity faculty because its logical foundation rests on establishing two distinct mental categories, creative and noncreative. Based on these two groups, stimuli, or conditions, a series of statistical contrasts is performed and the results are discussed. As the explicit intention is to separate out *the* creative dimension distinguishing the two mental classes, this paradigm, by design, controls for all cognitive and emotional processes known to operate in the mind, such as, for instance, fear, working memory, perception, theory of mind, top-down attention, emotional regulation, or a dozen other well-defined processes. As a matter of consequence, this experimental approach inherently leads to the investigation of creativity as a sovereign mental faculty.

To briefly anticipate a later discussion, the alternative approach, of course, would be to fully dissolve this mental unit into the same mental processes with which cognitive psychology conceptualizes and operationalizes all other higher-order mental faculties (Dietrich, 2019b). In contrasting these approaches further, the creativity faculty assumption essentially asks the question of what creative cognition is, and nothing else. In holding all other thinking processes constant, it necessarily looks for a specialized mechanism that integrates noncreative input and computes uniquely creative output. The alternative approach would fully collapse creativity into the well-known and well-defined units of cognitive psychology until it disappears into the brain’s standard information-processing system. There would be no residue containing or being creativity itself; there would be no further, specialized thing to find.

Similar thinking exists for the construct of consciousness. In the study of consciousness, this is best illustrated by the distinction between the easy and the hard problems (Chalmers, 1996). Following one line of argument, the easy problems are mental phenomena—attention, emotion, memory etc.—that lend themselves to scientific inquiry and are thus solvable in principle. In contrast, there is a hard problem—consciousness itself—which is something else, a thing unto itself. This problem cannot be solved, not even in principle, using the scientific method, since it is of a different kind. Nearly all of the famous thought experiments in the study of consciousness—philosophical zombie, qualia, Chinese room, Mary the color scientist, etc.—revolve around the assumption that consciousness is a discrete and separable thing (Blackmore, 2005; Dietrich, 2007b). Naturally, this position raises the question of what the nature of this further thing is. The opposing view is that consciousness cannot be separated from other mental processes. The hard problem is simply a collection of easy problems that solves itself as we make progress on these easy problems. By breaking down consciousness into its components from the outset and distributing it throughout the information-processing system, the hard problem, as a separate and separable entity, disappears (Dennett, 1996).

Since its introduction and critique (e.g., Fodor, 1983; Churchland, 1988), the concept of modularity is well established in psychology and neuroscience. A broad consensus has emerged in psychology holding that abstract, higher-order, and complex psychological constructs do not exist as discrete, monolithic cognitive faculties. While memory, attention, or perception remain common units of folk psychology, cognitive psychologists have long broken them up into types and subcomponents and developed empirical methods to investigate them. For instance, Posner and Petersen (1990) break up attention into several separate processes, such as engaging, disengaging, and shifting, while Baddeley (1986, 2000) divides working memory into the phonological loop, the visuo-spatial sketchpad, the episodic buffer, and the central executive. Accordingly, experimental research is no longer set up to study such folk psychology concepts directly and in their entirety.

This has not happened in the case of creativity. The initial idea of divergent thinking, for instance, has never been developed further. It is a vague compound construct, and no one, since its conception over half a century ago, has made a coherent proposal of what is in it in terms of the kinds of individual mental processes for which psychology has valid psychometric tools and that can be detected by neuroimaging technology, such as top-down attention, semantic retrieval, inhibition, shifting, cognitive control, spreading activation, feature detection, or a dozen other well-defined processes. Divergent thinking simply serves today, as it did then, as a stand-in for (the whole of) creativity, and the rationale and makeup of the testing instruments as well as the testing procedures elevate it to a sovereign cognitive entity in its own right and with its own boundaries. Creativity, therefore, continues to be treated, in theory and practice, as a standalone and monolithic faculty.

## 3 The creativity faculty in the brain

A key factor that has been kept clamped so far is the level of description. The degree of validity of a construct as an explanatory tool depends on the level of description at which it attempts to function (Bechtel, 2009). Creativity is commonly defined and measured not in terms of a mental event but of a product—*something* novel and useful (Runco, 2004) to which “surprising” is sometimes added (Boden, 2004). A patent office deems creative inventions, a buyer appraises paintings, an audience evaluates performances, and the Nobel Foundation awards scientific discoveries. The idea that there is a capacity or mental process that goes along with the creative product is an abstract and hypothetical construct. This is uncontroversial psychology, along with the consensus that there is a near endless number of ways humans can produce a creative product.

The matter becomes a fallacy when the abstract and hypothetical construct is mistaken for a real and definite entity. This fallacy of concreteness and illusionary unity is known as the reification fallacy (e.g., Boag, 2011). Reification is the process of taking an ambiguous and abstract concept and turning it into a tangible and concrete thing. As can be seen by the claims about (the whole of) creativity cited in the beginning of this paper as well as the empirical methodology of establishing a categorical distinction between creative and noncreative, “thing-making” is the default paradigm of creativity research. And once in place, researchers tend to ignore the complexities contained in the concept and begin to treat it as a homogeneous whole.

At the macroscopic level of human interactions in society, the folk conception of creativity as a discrete and holistic psychological entity might have some utility. Even if reified, a creativity faculty can still meaningfully inform a research program on creativity—at that level. Some might argue, therefore, that reification can be tolerated at that level of description, if the limits of the construct remain properly conceptualized.

But this fallacy becomes deadly when it is applied, *tout court*, to lower levels of description. Departing from a reified, *bona fide* creativity faculty, cognitive neuroscientists have simply adopted the invalid, but readily available, testing methodology used in cognitive psychology and added a physiological measurement as a dependent variable to it, such as EEG or fMRI. This has led to the same, direct creative-*vs*-noncreative contrasts, except that such a brain study inherently looks for a one-to-one match between the creativity faculty and the neurocognitive substrates exclusively dedicated to it. After all, creative thinking is obviously special and there must be *something* that makes it so.

It is helpful to flesh out what this course of action commits us to at the level of the brain. In both rationale and methodology, it assumes, from the outset, the existence of uniquely creative cognition—something specific in the brain that unites creative behavior across all the widely different domains, contexts, and instantiations, from a modern dance performance to ideas in quantum physics. At the cognitive level, a creativity faculty would imply some sort of special cognitive process or processes not associated with any other mental capacity. It could also be the computation itself, the set of rules and permissible transformations that is supposed to perform the specialized creative computation. At the neural level, this mapping would imply some sort of corresponding marker of brain activity that manages *only* creativity and *all* creativity.

Given that such a research program violates the consensus view that creativity is complex and diverse, and thus supported by many different cognitive and emotional processes housed all over the brain, it is not surprising that a creativity faculty has so far neither appeared anywhere in the brain, nor are there any coherent theoretical proposals for what its nature might be.

The fallacy of a standalone creativity faculty in the brain can perhaps best be seen by drawing on the historical parallel of phrenology (Dietrich, 2015). In the 1800s, Francis Gall associated 27 mental faculties with areas on the skull, including centers for mirthfulness, combativeness, marvelousness, secretiveness, and the organ of philoprogenitiveness (love for offsprings). The error comes into clear view here because modern personality theory no longer considers these psychological constructs entities. And, with reification exploded, the fallacy of a matching unit at the cognitive or neural levels of description is also laid bare. Secretiveness simply does not refer to a real thing in the brain.

A curious disconnect in the field is that, once drawn out like this, creativity neuroscientists typically waste no time explicitly renouncing any allegiance to a creativity faculty in the brain. Undoubtedly as a result of the association with phrenology, they would climb over one another to put ideological distance between themselves and this fallacy. Nevertheless, virtually any study in the cognitive neuroscience literature betrays a commitment to it, both in terms of rationale and methodology (for a few representative examples, see Carlsson et al., 2000; Howard-Jones et al., 2005; Takeuchi et al., 2010; Baird et al., 2012; Benedek et al., 2014; Fink and Benedek, 2014; Kounios and Beeman, 2014; Green et al., 2015; Beaty et al., 2016).

**Figure 1:**
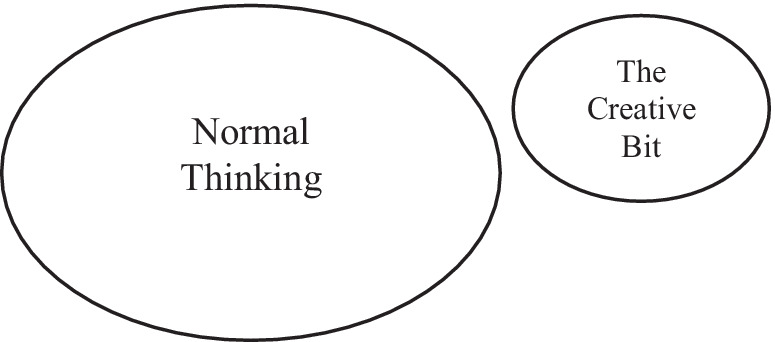
The creative bit. A creativity faculty in the brain that manages *only* creativity and *all* creativity. For the current neuroimaging paradigm to make sense, this entity (1) must exist as a separate and separable thing, (2) be extractable by the psychometric method, (3) have a proprietary neural code that (4) is visible to the neuroimaging technology we currently have. For creativity, none of this probably exists.

Indeed, the entire rationale of current neuroimaging studies of creativity rests on the premise that there actually is such a thing as a creativity faculty and that this thing exists, as such, in the brain. Logically inherent in this rationale is that there must be some sort of contrasting “normal” thinking occurring in the brain—the control condition, in other words—to which an extra something—the creative bit—is added to make the sparkling difference. Looking for *it* in the brain just makes plain sense (notice the singular). With the creativity faculty firmly in place, the experimental procedure is then deliberately designed with the intention of isolating this creative bit and detecting it with neuroimaging tools. It does so by making a series of direct creative versus noncreative contrasts with the goal of identifying (1) uniquely creative cognition that is distinct from all other kinds of cognition and that (2) has an exclusive neural signature, be it a brain region, a neural network, connectivity, or any other substrate system proprietary to creativity (Figure 1).

Even if we bracket for a moment the mountain of evidence showing that the current psychometric instruments in the field, such as the Alternative Uses Test, do not possesses any demonstrated ecological validity (for a detailed critique, see Baer, 2022), any finding from this neuroscience paradigm should be considered an artifact of misguided theorizing. It simply follows from the complexity and diversity inherent in the psychological construct of creativity that there must be a multitude of very different, and perhaps even opposing, mental processes and neural instantiations that can possibly result in the creation of something novel and useful. But as the sweeping generalizations in the opening statements show, it is one thing to commit to a view and quite another to go along with all the consequences that come with it. When we factor in complexity, as we must, we really have to factor it in.

Again, other areas of cognitive neuroscience such as attention or memory can serve as examples of good practices (Barrett, 2009; Satel and Lilienfeld, 2013). The folk concept of memory, for instance, has undergone a radical conceptual change throughout the twentieth century and today no cognitive neuroscientist would present a neuroimaging study on (the whole of) memory or draw conclusions mirroring the quotes from the beginning of this paper. Indeed, types or subcomponents of memory, such as episodic memory and working memory, are decomposed ever further with ever more specific cognitive tests, and there are multiple meta-analyses showing cumulative and congruent insights (e.g., Chen et al., 2017; Emch et al., 2019).

In a more recent example, moral neuroscience shifted soon after its founding from a moral faculty assumption and a direct moral-*vs*-nonmoral experimental approach to investigating the contribution of standard mental processes to moral judgment (Young and Dungan, 2012). That is, rather than controlling for nonmoral dimensions in an experiment, researchers studied them directly, by, for instance, comparing different types of moral stimuli in order to discern which mental processes and neural substrates play a role in which type of moral reasoning.

## 4 The false category formation

The first error in the statements at the beginning of the article is the creativity faculty fallacy, which results from the failure to decompose creativity into the information-processing units that make up the standard knowledge base of psychology and neuroscience. The second error is the false category formation. The latter error results from the failure to carve nature at its joints. It occurs subsequent to the creativity faculty fallacy and is committed by ascribing to (the whole of) creativity one pole of various bipolar dimensions, ignoring the other end. Indeed, it is the second error, the false category formation, that fully exposes the first error, the creativity faculty fallacy, because without this second step, the reification process underlying the “thing-making” of creativity would be difficult to detect otherwise.

Divergent thinking serves as the canonical example. By common consent, which is also now reflected in items of the TTCT (Cramond and Kim, 2009), one can also be creative with the exact opposite process: convergent thinking (Weisberg, 1999; Runco, 2004; Dietrich, 2007a; Simonton, 2015). Standard examples might be Edison’s “empirical dragnet” method that yielded a total of 1,093 patents; Watson and Crick’s disciplined approach of testing the stability of DNA base pairs; Bach’s assembly-line tactic of composing hundreds of cantatas; or the countless times we converged on creative solutions by methodically eliminating alternatives?

This raises the obvious question of what, exactly, is creative about divergent thinking. If both divergent and convergent thinking can lead to both creative and non-creative thinking, the concept of divergent thinking is incapable of isolating the subject matter of interest – creativity! In short, the use of divergent thinking as a proxy for creative thinking is theoretically incoherent. The treatment and the control condition cannot contain the same variable.

Like the creativity faculty fallacy, the false category formation is ubiquitous. A selected list of the most common claims of what is supposed to be associated with (the whole of) creativity might be divided into processes (divergent thinking, defocused attention, latent inhibition, intelligence, imagination, intuition, remote associations, lateral thinking, cognitive dissonance, incubation, etc.), states of consciousness (REM sleep, madness, daydreaming, mindfulness, psychedelic drugs, flow, unconscious thinking, etc.), or brain activity (right brains, prefrontal cortex, low arousal, alpha synchrony, default mode network, network connectivity, etc.) (Mednick, 1962; DeBono, 1968; Singer, 1975; Jamison, 1993; Martindale, 1995, 1999; Schooler and Melcher, 1995; Boden, 1998; Carlsson et al., 2000; Pfenninger and Shubik, 2001; Carson et al., 2003; Wagner et al., 2004; Howard-Jones et al., 2005; Dijksterhuis, 2006; Baird et al., 2012; Jung et al., 2013; Fink and Benedek, 2014; Kounios and Beeman, 2014; Green et al., 2015; Beaty et al., 2016; Chrysikou, 2018; Nikolaidis and Barbey, 2018).

Paradoxically, it is uncontroversial in the field that their opposites—focused attention, “ordinary” consciousness, convergent thinking, for instance—or their invariability can also be sources of creative behavior (e.g., Weisberg, 1999; Dietrich, 2015; Simonton, 2015). In addition, all these processes, states of consciousness, and brain activity pattern are also involved in non-creative cognition. The default mode network, for instance, has been linked to nearly everything by now from stimulus-independent thought, stimulus dependent thought, social cognition, mind-wandering, or self-referential thinking (e.g., Legrand and Ruby, 2009). Regardless of how strongly the association to creativity might feel, processes such as intuition, incubation, insight, divergent thinking, or remote association can produce non-creative outcomes, reinforcing the conclusion that such simple, *a priori* divisions are instances of false category formations.

The false category formation is a powerful illusion. Such quick and simple associations seduce us into thinking that we have done all the theorizing work that can be done, that we can stop, when, in fact, we have solved nothing. An easy way to break the back of this comforting but misleading habit is to simply ask ourselves a few straightforward, follow-up questions each time we fall prey to it: What, exactly, is creative about it? How is creativity linked to, say, intuition? For what kinds of creativity might the opposite be true? In which situations does it not apply? What domains are included, which excluded? Since the associated phenomenon also plays a role in noncreative thinking, what aspect of it makes it uniquely creative? What such an exercise would reveal is that, upon further inspection, virtually no claim survives closer scrutiny if applied to creativity as a whole. This should tell us, in turn, that we need to go back to work on our understanding of creativity.

The lack of such a line of probing questions is likely also the reason why falsification has failed in the field. For any one claim, instances of creativity are easy to find, and, without a follow-up, none is ever retired or leads to a decomposition of the multifaceted concept of creativity. This has generated a highly fragmented literature (e.g., Arden et al., 2010; Dietrich and Kanso, 2010; Sawyer, 2011; Yoruk and Runco, 2014; Wu et al., 2015; Abraham, 2018; Cogdell-Brooke et al., 2020). To give two examples of this deeply self-contradictory literature, creativity—again, without qualifying it any further—is said to be associated with mental disorders (e.g., Jamison, 1993; Kaufman, 2005), a claim that lives happily side by side in the literature with its opposite, that is, creativity is accompanied by psychological wellbeing (e.g., Csikszentmihalyi, 1996; Dietrich, 2014) and positive thinking (Seligman, 2002). Creativity is also said to be linked to low arousal (Martindale, 1999), which cannot be reconciled with creative acts in high-pressure situations, such as the imaginative ways in which NASA engineers solved the problems of the otherwise doomed Apollo 13 mission or a creative move in the last seconds of a basketball game to beat the buzzer. One can do this back-and-forth with nearly all claims, including, for example, attention, intuition, playfulness, working memory, happiness, intelligence, inhibition, incubation mindwandering, states of consciousness, drug states, prefrontal cortex activation, or dopamine.

In sum, the one-two punch of the creativity faculty fallacy followed by the false category formation virtually ensures that statements such as those at the beginning of the present paper are false. Given the sheer varieties and complexities, in domains and processes, in which humans can possibly generate creative products, any association of creativity, as a reified and monolithic unit, to any phenomenon is likely to be false, irrespective of what specific claim is being made. Creativity *per se* simply cannot be linked to any trait, thing, characteristic, behavior, habit, mental process, or neural activity in a straightforward manner.

## 5 What if creativity is fully integrated in the brain?

Naturally, this leaves open the question of how else we should think about creativity. Creative achievements are so remarkable that there must be a striking difference, a place or mechanism that marks the crucial moment when a creative idea jumps out from all the noise of the ordinary mental buzz. The creativity faculty is such an intuitive way of thinking about creative cognition that the fallacy is nearly impossible to shake. But an examination of this intuition shows that it cannot find a defensible position within the information-processing theories of psychology and neuroscience. Like other complex, higher-order psychological phenomena—political conviction, goodness, or religious belief, for instance—creativity does not exist as its own, specialized entity at the cognitive or neural levels, despite seeming so at the psychological level.

A more capable candidate is the conception that the neurocognitive mechanisms of creativity are distributed, embedded, and varied; that is, creativity is everywhere and multiply realizable by the standard functional units of cognitive neuroscience (Dietrich, 2015). The position follows, as a matter of consequence, from two basic concepts in neuroscience—nonlinearity and modularity.

Nonlinearity refers to the understanding that the brain is a dynamic information processor. In consequence, every neural circuit or network that computes information must also produce novel combinations, or variation, of that information. Indeed, novelty is an inevitable outcome of a complex, nonlinear system. Novelty production, then, is distributed in the brain (Dietrich, 2004).

Modularity refers to the understanding that the brain is organized into specialized modules. In consequence, neural networks that process specific content to yield “normative” combinations of that content must also be the neural networks that generate creative combinations of that content. That is, the recombination of bits and pieces of content into novel configurations must come from the same neural circuits that normally handle those bits and pieces of content. The assumption of a further, independent module whose output is separately added to a neural circuit to render its computation creative, makes neither computational sense nor has any evidentiary basis. Creative computation, then, is embedded (Dietrich, 2004). If painting, math, and parking the car engage totally different brain areas and processes, so should creative painting, creative math, and creative car-parking. Creativity, in this view, is not a separate and separable thing in the brain, but an emerging outcome of the brain’s standard information-processing operations.

Finally, multiple realizability refers to the idea that creativity can be realized by a wide variety of standard mental processes, properties, states, events, neural mechanisms, or their combination. Indeed, the combinatorial possibilities might be vast. For instance, some creative products might come about in a state of low arousal, while others can be generated by high arousal or, indeed, no change in arousal levels at all. Likewise, some creative thinking might require the engagement of focused attention, while other forms might benefit from more mindwandering. Yet others need perhaps more episodic memory, or fine motor skills, or more acetylcholine transmission, or a cognitive restructuring. In short, there is likely an innumerable array of coordinated patterns at several levels of the functional system of the brain that could support the computation of a novel, useful, and perhaps surprising outcome. And this is not even accounting for the fact that real-world creative behavior is temporarily extended, requiring many different steps, each requiring very different processes and substrates, before a creative end product sees the light of day.

There is neuropsychological evidence that is consistent with the idea of multiple realizability. For instance, there are several studies showing that brain damage does not impair creativity, with artists continuing to be creative despite relevant brain impairments (Bogousslavsky, 2005, 2006). Similarly, using the arts (e.g., melodic intonation) for rehabilitating patients with aphasia points toward multiple routes for creativity (Norton et al., 2009).

Fully disciplined, this conception on the neural basis of creativity is bad news for a localizationist position and/or uniqueness assumption on creativity. If creativity, as argued here, is not its own specialized thing at the cognitive or neural levels, but distributed, embedded, and multiply realizable by the brain’s ordinary functional components, what the current neuroimaging template based on the creativity faculty assumption would find, depends only on how we decide to look. Besides there being no uniquely creative cognition or neural code, each purported “creativity test” would only implicate its own, idiosyncratic set of cognitive processes and neural activity. While the description of one such pattern might still be a worthwhile end, it would have no bearing on the next “creative task,” let alone apply to creativity as a whole. Bracketing again the lack of test validity for a moment, this inference is supported by the highly variegated results of the field (e.g., Dietrich and Kanso, 2010; Sawyer, 2011). More recent meta-analyses based on activation likelihood estimation (ALE), if taken together (e.g., Boccia et al., 2015; Wu et al., 2015; Pidgeon et al., 2016; Cogdell-Brooke et al., 2020), also show this absence of the cumulative character that is so impressive in other fields of cognitive neuroscience, such as (the various processes comprising) attention (Posner and Petersen, 1990), memory (Gazzaniga and Mangun, 2014), or morality (Young and Dungan, 2012).

## 6 Recommendations

How, then, should the neuroscience of creativity proceed? How should we rethink our experimental approach to creativity? Here are some recommendations that arise from adopting a conception of creativity as fully integrated into the brain’s information-processing operations.

First is theory. In a view approaching unanimity in the field, the concept of creativity needs further theoretical development. But one could hardly conclude that there is a necessity for theoretical development from a perusal of the literature. Even when investigators acknowledge the problem in their introductory remarks, studies proceeds, as if the acknowledgment alone turns the water into wine, using the same basic rationale and methodology that is essentially unchanged since Guilford’s efforts in the 50s and 60s. Obviously, the interpretations of the findings from this divergent-thinking-test-plus-neuroimaging paradigm have kept pace with current knowledge in cognitive neuroscience—default mode network or connectivity—but that is not the side of the equation that needs change and theory development. Einstein once remarked to Heisenberg (as cited by Fullbrook, 2012, p. 20) “Whether you can observe a thing or not depends on the theory you use. It is theory which decides what can be observed.”

A neuroscience study claiming to present findings on creativity *per se*, even if the creativity faculty assumption is overtly denied by the authors, will most certainly qualify as phrenology. Creativity neuroscientists cannot run a study on creative thinking any more than cognitive psychologists and neuroscientists can run a study on thinking. Global statements about the nature of (the whole of) creativity of the kind that opened the present paper are illusion generators.

Second are types. In being more specific about theory development, the obvious way to start making the neurocognitive mechanisms of creativity more tractable is to parse creativity into different subtypes. One recent effort (Dietrich, 2015, 2019b) proposes to divide creativity into three distinct types, a deliberate mode, a spontaneous mode, and a flow mode. To avoid the pitfalls of previous such attempts, the three creativity types are explicitly defined and delineated from one another based on the standard units of cognitive psychology and neuroscience. By anchoring them in the existing knowledge base, they are thus valid types in the sense that they can be theoretically defended. A division into three subtypes can only be regarded as a start, however. Eventually, creativity would decompose fully into the same functional components that we use to operationalize all other complex, higher-order mental capacities, such as attention, memory, or morality.

Third is approach. The integration conception of creativity defeats a neuroscientific research program that is based on investigating (the whole of) creativity directly, in a bipolar, yay-nay fashion. If a neuroscience experiment is set up along the dimension of creative vs. noncreative and a series of contrasts on the generated data set is performed, the study unequivocally looks, as a matter of consequence, for the brain’s *creative bit*, regardless of whether this proprietary entity is a still undiscovered substrate or a unique pattern of known ones. Inherent in this method is to treat all other cognitive and emotional processes as confounds, which are thus held constant. In other words, the mental processes that constitute the bedrock of cognitive neuroscience are not seen as independent variables; they are only used as outputs, or dependent variables. As stated above, advances in neuroscience in recent decades have only been used to interpret the findings from this paradigm; they have not been used to contribute to the theoretical development, or breakdown, of the concept of creativity itself. Accordingly, experiments in creativity research should treat all the standard cognitive and emotional processes of cognitive neuroscience as likely inputs to (different types of) creative cognition. Developments in the neuroscience of memory and, more recently, morality can serve as instructive examples for the future direction of the neuroscience of creativity.

## Data availability statement

The original contributions presented in the study are included in the article/supplementary material, further inquiries can be directed to the corresponding author.

## Author contributions

AD: Writing – review & editing, Writing – original draft, Conceptualization.
